# BMP-induced reprogramming of the neural retina into retinal pigment epithelium requires Wnt signalling

**DOI:** 10.1242/bio.018739

**Published:** 2017-05-25

**Authors:** Jörg Steinfeld, Ichie Steinfeld, Alexander Bausch, Nicola Coronato, Meggi-Lee Hampel, Heike Depner, Paul G. Layer, Astrid Vogel-Höpker

**Affiliations:** Fachbereich Biologie, Abteilung Stammzell- und Entwicklungsbiologie, Schnittspahnstraße 13, Darmstadt 64287, Germany

**Keywords:** Cell fate, Progenitors, Retinal pigment epithelium, Regeneration, Retina, Stem cells, Transdifferentiation, Re-specification

## Abstract

In vertebrates, the retinal pigment epithelium (RPE) and photoreceptors of the neural retina (NR) comprise a functional unit required for vision. During vertebrate eye development, a conversion of the RPE into NR can be induced by growth factors *in vivo* at optic cup stages, but the reverse process, the conversion of NR tissue into RPE, has not been reported. Here, we show that bone morphogenetic protein (BMP) signalling can reprogram the NR into RPE at optic cup stages in chick. Shortly after BMP application, expression of *Microphthalmia-associated transcription factor* (*Mitf*) is induced in the NR and selective cell death on the basal side of the NR induces an RPE-like morphology. The newly induced RPE differentiates and expresses *Melanosomalmatrix protein 115* (*Mmp115*) and RPE65. BMP-induced *Wnt2b* expression is observed in regions of the NR that become pigmented*.* Loss of function studies show that conversion of the NR into RPE requires both BMP and Wnt signalling. Simultaneous to the appearance of ectopic RPE tissue, BMP application reprogrammed the proximal RPE into multi-layered retinal tissue. The newly induced NR expresses *visual segment homeobox-containing gene* (*Vsx2*), and the ganglion and photoreceptor cell markers Brn3α and Visinin are detected. Our results show that high BMP concentrations are required to induce the conversion of NR into RPE, while low BMP concentrations can still induce transdifferentiation of the RPE into NR. This knowledge may contribute to the development of efficient standardized protocols for RPE and NR generation for cell replacement therapies.

## Abstract (precis)

**Summary:** The bone morphogenetic proteins (BMPs) are key regulators in cell reprogramming during regeneration of the neural retina and retinal pigment epithelium in the embryonic chick.

## INTRODUCTION

In vertebrates, the retinal pigment epithelium (RPE) is a single-layered pigmented epithelium that supports metabolic and cellular processes of the light-sensitive photoreceptors located in the multi-layered NR (reviewed in Strauss, 2005, 2011). Many sight-threatening diseases are caused by RPE or NR degeneration, and treatments for these blinding diseases are still limited. The RPE also exerts important roles during eye development. For instance, isolated RPE cells from the eye periphery of the embryonic day (E)5 chick embryo supported *in vitro* formation of completely laminated 3D retinal spheroids, establishing that retinal tissue can be produced *in vitro* by self-organisational processes recapitulating normal retinogenesis (Layer and Willbold, 1989, 1994; Layer et al., 2001). Recently, cell replacement of the lost RPE or NR has become a primary strategy in the field of regenerative ophthalmology. Currently, pluripotent stem cells, i.e. embryonic stem cells or induced pluripotent stem cells, are used to recapitulate normal developmental processes to generate RPE and NR (reviewed in Sasai, 2013, Sasai et al., 2012; Ramsden et al., 2013; Viczian, 2013; Fuhrmann et al., 2014; Martinez-De Luna and Zuber, 2014; Reynolds and Lamba, 2014; Leach and Clegg, 2015). For example, an efficient method for deriving a functional RPE cell population from both human embryonic and induced pluripotent stem cells has recently been described (Choudhary et al., 2017). Here, the sequential inhibition and activation of the activin and bone morphogenetic signalling pathways (see below) allowed the directed differentiation of a homogenous and functional RPE population (Choudhary et al., 2017). Besides this, nutrition has been shown to play an important role in preventing blindness in patients with age-related macular degeneration (reviewed in Campbell and Campbell, 2004).

During vertebrate eye development, extrinsic signals released from the surrounding ocular tissues pattern multipotent optic vesicle cells into an RPE or NR domain. The determination of optic vesicle cells towards a NR or RPE cell fate involves multiple steps. The earliest restriction of optic vesicle potency from the multipotent condition occurs when these cells become specified into *visual segment homeobox-containing gene* (*Vsx2*)-expressing NR, or *Microphthalmia-associated transcription factor* (*Mitf*)-expressing RPE progenitor cells. At this stage of specification, cell commitment is still reversible, i.e. the cell can still be respecified or transformed into another cell type. The next step towards differentiation is when the cell becomes determined towards a certain cell type. At this stage, the cell differentiates autonomously even when placed in a non-neutral environment. Transplantation and ablation experiments are an excellent way to determine if a cell is specified or determined. For example, following surface ectoderm removal at stage 9 in the chick, *Mitf* expression is lost and consequently RPE development is not observed (Steinfeld et al., 2013). In contrast, only a few hours later *Mitf* expression in the distal optic vesicle is stable, and signals released from the overlying surface ectoderm are no longer required (Hyer et al., 1998; Nguyen and Arnheiter, 2000; Kagiyama et al., 2005; Steinfeld et al., 2013). Thus, at stage 9, chick optic vesicle cells are specified to develop into RPE and this fate appears to be determined by stage 10 (>11 somites). However, once a cell has differentiated it still can be converted from one cell type into another; a process that is called transdifferentiation. In several species, conversion of the RPE into NR can be induced by extrinsic or intrinsic signals (reviewed in Spence et al., 2007b; Araki, 2007; Barbosa-Sabanero et al., 2012; Martinez-De Luna and Zuber, 2014; Pittack et al., 1991; Spence et al., 2007a; Luz-Madrigal et al., 2014). In the chick, the RPE can only be induced to develop into a well-layered NR in the presence of fibroblast growth factors (FGFs) or upon *Pax6* overexpression (Park and Hollenberg, 1989, 1991; Pittack et al., 1997; Azuma et al., 2005). Two major strategies are used to convert tissues of the eye in vertebrates (reviewed in Spence et al., 2007b; Martinez-De Luna and Zuber, 2014). In the chick optic cup, one strategy to induce ectopic NR involves the activation of stem or progenitor cells located in the peripheral region of the eye, the ciliary margin (CM), whereas the other strategy to induce ectopic NR involves the transdifferentiation of RPE cells in the outer layer of the optic cup. In the chick this capacity is lost around E5; however, when these cells are placed in culture or when *Pax6* is overexpressed in the chick RPE, the capacity to induce ectopic NR is still retained up to E9-E12 (Willbold and Layer, 1992; Azuma et al., 2005). Whereas the RPE can be induced by growth factors to develop into NR *in vivo*, the conversion of NR tissue into RPE has only been observed *in vitro* (Araki and Okada, 1977, 1978; Okada et al., 1979; reviewed in Araki, 2007; Belecky-Adams et al., 2008). During chick eye development, both the BMP and Wnt signalling pathways are required to initiate the RPE-inducing gene *Mitf* and hence RPE development (Steinfeld et al., 2013; Pandit et al., 2015), whereas BMP4 has been shown to be involved in NR specification in both mouse and chick at optic vesicle stages (Huang et al., 2015; Pandit et al., 2015). The function of BMPs at later stages (optic cup stages), has already been investigated in chick and mouse (Murali et al., 2005; Behesti et al., 2006; Trousse et al., 2001; Haynes et al., 2007). Here, we extend these studies and show, for the first time, that BMP5 signalling can induce ectopic RPE development in retinal cells of the chick optic cup *in vivo*. This tissue conversion requires both high BMP concentrations and Wnt signalling. Moreover, our results suggest that BMP signalling selectively induces apoptosis in retinal cells at optic cup stages, thereby allowing the appearance of an RPE-like morphology. Lastly, we provide novel data that BMP signalling can also reprogram proximal RPE cells to develop into NR.

## RESULTS

### *Bmp5* is expressed in the developing RPE and in surrounding tissues at optic cup stages

**Fig. 1.**
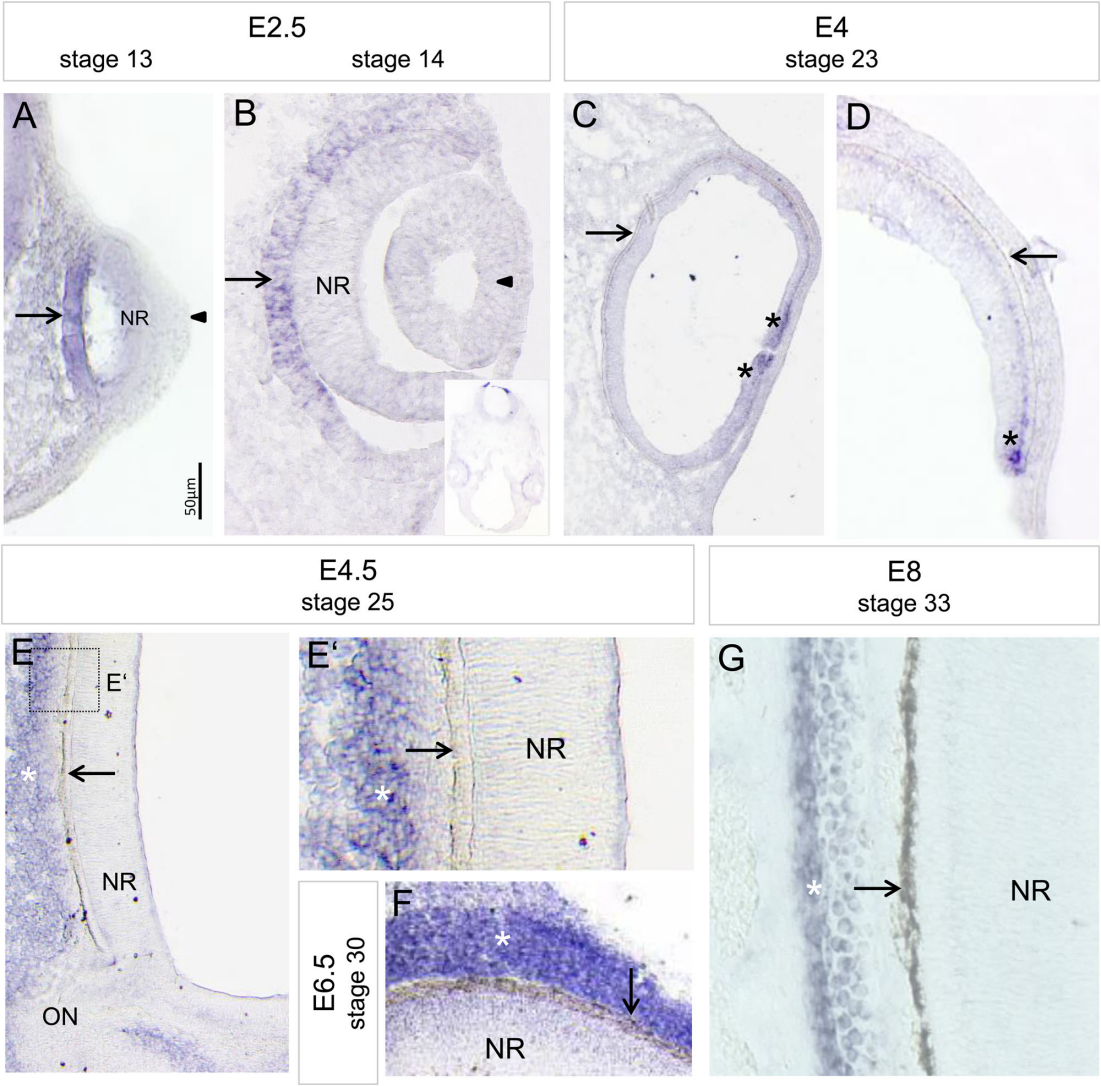
***Bmp5* expression pattern during chick eye development.** (A,B) *Bmp5* expression is detected in the presumptive RPE at stage 13/14 (E2.5; arrows). While no *Bmp5* transcripts are detected in the lens placode/vesicle at these stages (arrowheads), faint *Bmp5* expression appears to be in the presumptive NR at optic vesicle stages (stage 13). The inset in B shows the complete naso-temporal section of the head at stage 14. Note that *Bmp5* expression is faint within the presumptive RPE when compared to the expression found in the roof plate of the hindbrain region (rhombomere 1). (C,D) At stage 23 (E4), *Bmp5* expression is faint or absent in the proximal and peripheral RPE (arrows), while *Bmp5* transcripts are detected at the optic cup rim (asterisks). (E,F) At stages 25 to 30 (E4.5-E6.5), strong *Bmp5* expression is detected in the mesenchyme surrounding the RPE (white asterisks), while no *Bmp5* transcripts are detected in the RPE (arrows). (G) At stage 33 (E8), *Bmp5* transcripts are detected adjacent to the RPE (arrow), in the tissues which will develop into the choroidea and sclera (white asterisk). All expression pattern studies included *n*≥3 per stage.

The dynamic expression pattern of *Bmp2*, *-4* and *-7* has been extensively analysed during mouse and chick optic vesicle and cup stages (Fig. S1) (see Discussion). Here, we investigated the exact expression pattern of the BMP-family member, *Bmp5*, at late optic vesicle and cup stages in the chick (stage 12-33). The first *Bmp5* transcripts were detected in the presumptive RPE at stage 13 at the time when the lens placode forms (Fig. 1A). At stage 14, *Bmp5* expression was detected in the presumptive RPE with transcripts being more abundant in the proximal region. *Bmp5* expression was also observed in the mesenchyme at the optic cup margin (Fig. 1B; Fig. S1). Expression of *Bmp5* persisted in the RPE until atleast stage 19 (Fig. S1). *Bmp5* expression appeared now to be stronger in the ventral RPE when compared to dorsal RPE, whereas mesenchymal expression appeared to be stronger dorsally. At later stages, weak *Bmp5* expression was still observed in the peripheral eye, whereas expression was strongly detected in the tissue (presumptive choroid and/or sclera) adjacent to the RPE (Fig. 1C-G; Fig. S1). Thus, the observed expression patterns indicate that BMP signalling is still involved in RPE development at optic cup stages.

### BMP5 signalling induces hyperpigmentation and ectopic RPE at optic cup stages

**Fig. 2.**
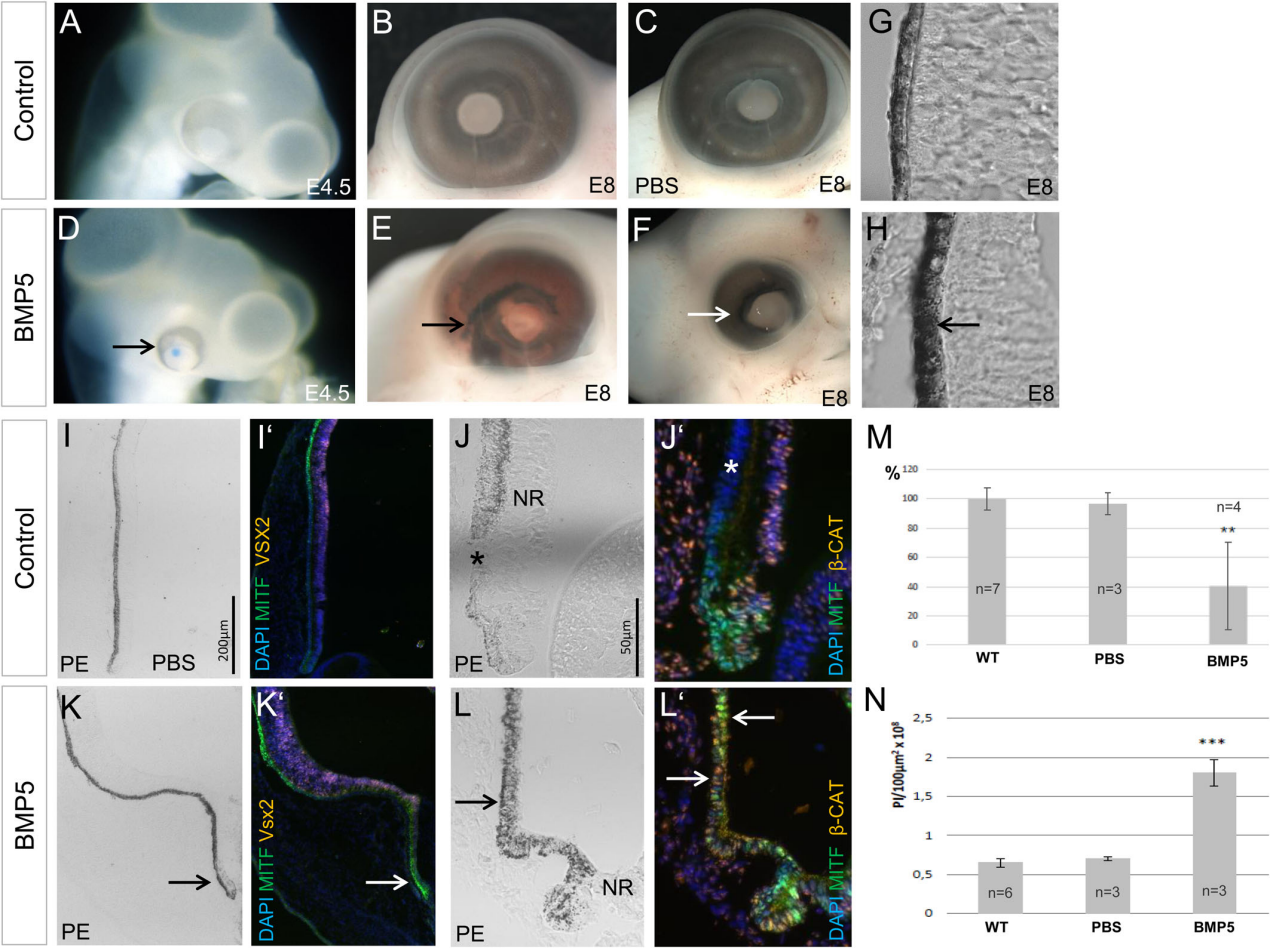
**BMP5 signalling induces hyperpigmentation of the chick RPE.** (A-F) Eye morphology and pigmentation of the contralateral (control), PBS- and BMP5-treated eyes at E4.5 and E8 following manipulation at E3/4. The BMP5-treated eyes show signs of hyperpigmentation (arrows) when compared to the controls (27/34). (G,H) Sections of the proximal eye of the contralateral (control) and BMP5-treated eye. The RPE of the BMP5-treated eye appears to be stronger pigmented (arrow) when compared to the untreated eye. (I-L′) Pigmentation, MITF and β-Catenin protein localisation at E8 in control and BMP5-treated eyes. Following BMP5 application, pigmentation appears to be stronger and ectopic MITF and β-Catenin protein is detected in the pigmented regions (arrows), when compared to the untreated eye (asterisks). Note that in L,L′ the inner layer is detached from the outer, pigmented layer of the eye. (M) Graphical representation of the percentage reduction in eye size following application of a PBS- or BMP5-soaked bead, when compared to the contralateral, untreated eyes (WT). A significant reduction in eye size was observed following BMP5-treatment (**P≤0.01), while the PBS-treated eyes showed no significant reduction in size when compared to the untreated eyes. (N) Quantitative analysis showing hyperpigmentation in untreated, PBS- and BMP5-treated eyes. There is a significant difference in pixel intensity after BMP5 treatment (***P≤0.001) but no significant difference following PBS treatment (*P*>0.05) when compared to the contralateral (control) eye. All expression pattern studies included *n*≥5 (Table S1). PE, Peripheral eye.

BMPs can induce *Mitf* expression and/or pigmentation in multipotent optic vesicle cells (Ohkubo et al., 2002; Hyer et al., 2003; Steinfeld et al., 2013). To investigate the effects of BMP signalling at later stages of eye development, we implanted BMP5-soaked beads at optic cup stages in the chick (E3.5/E4). Following bead implantation, hyperpigmented microphthalmic eyes developed in the majority of the cases 1-4 days after the operation (Fig. 2A-F,M; Table S1). Quantitative analysis showed that exposure to BMP5 resulted in hyperpigmentation in both the proximal and peripheral RPE (Fig. 2H,L,N). Moreover, strong nuclear MITF protein accumulation, normally only detected in the proximal RPE (Fig. 8D,E), was now detected in the peripheral region of the outer optic cup (Fig. 2I-L′). The Wnt/β-Catenin signalling pathway is involved in RPE development in vertebrates (reviewed in Fuhrmann, 2010; Fujimura, 2016). In the peripheral eye nuclear β-Catenin is detected in inner retinal cells but not in the pigmented peripheral eye margin at E3.5-E8 (Fig. 2J′ and Fig. 1C-G). BMP5 application resulted in nuclear β-Catenin accumulation in the hyperpigmented regions (Fig. 2L,L′). The development of microphthalmic, hyperpigmented eyes was not observed when PBS-soaked beads were implanted at the same stage (Fig. 2C,J,M,N; Table. S1) and MITF- and β-Catenin protein distribution was not affected in the peripheral eye (Fig. 2G). Taken together, these results indicate that BMP5 application induces hyperpigmentation and proximal RPE characteristics in the peripheral outer margin of the chick optic cup.

### BMP5 signalling reprograms retinal cells in the peripheral optic cup

**Fig. 3.**
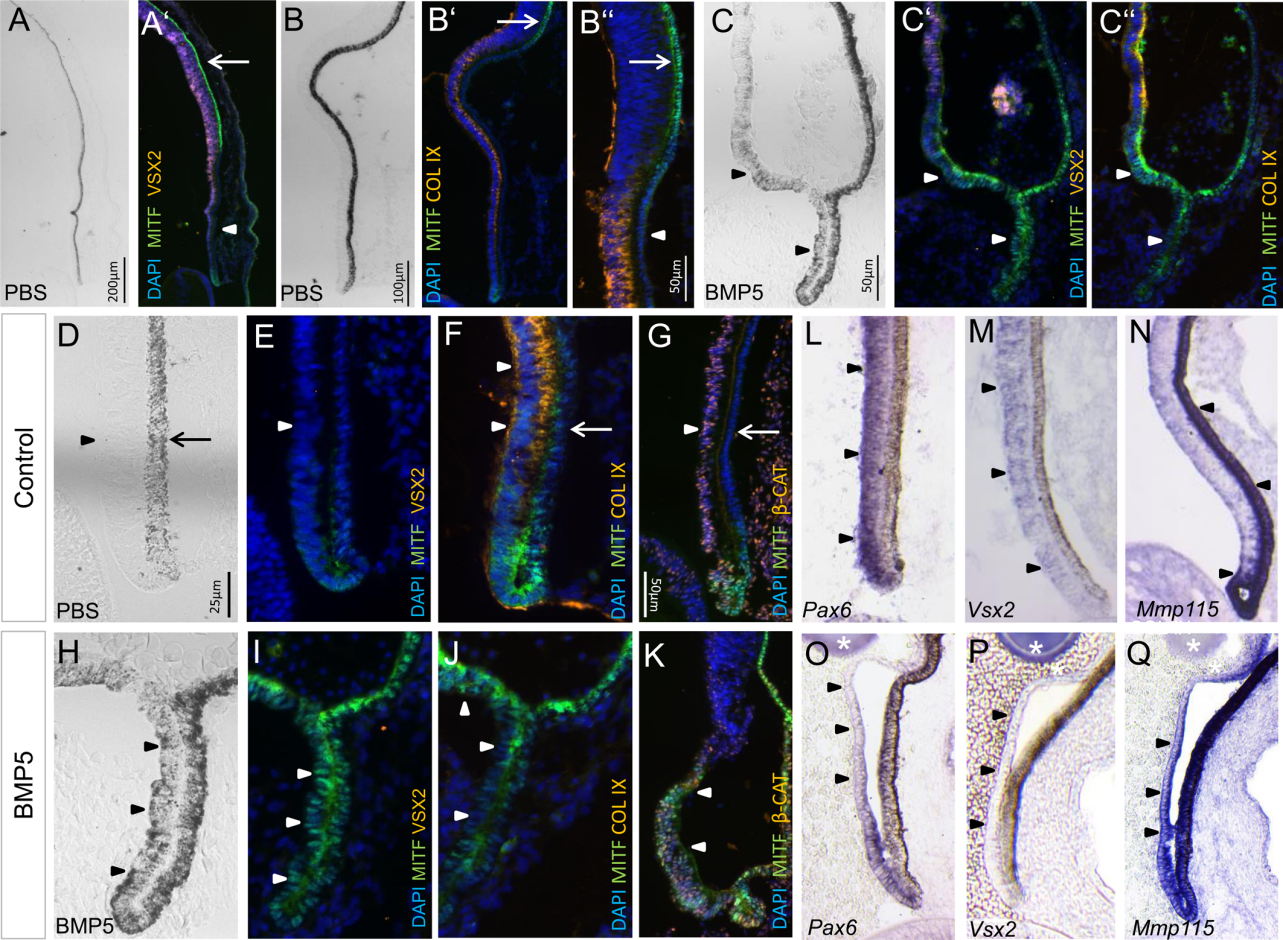
**BMP5 induces ectopic RPE in the peripheral region of the chick optic cup.** (A-B″) Pigmentation and distribution of MITF-, VSX2- and Collagen IX protein in the peripheral eye cup at E8. MITF protein is strongly detected at the optic cup rim and in the proximal MITF-positive RPE (arrows). Arrowheads indicate the peripheral RPE with faint or no MITF labelling. (C-C″) Following BMP5 application (0.7 µg/µl) at E3.5/4, ectopic pigmentation and MITF protein is detected in the NR, whereas VSX2 and Collagen IX protein is not detected at E8 (arrowheads). (D,E) Higher magnification of the peripheral eye cup at E8. Pigmentation, MITF and VSX2 protein is only weakly or not detected in the inner layer (arrowheads). The arrow in D indicates the RPE. (F,G) Collagen IX and nuclear β-Catenin is detected in the inner optic cup (arrowheads) and are not detected in the RPE (arrows). (H-K) Following BMP5-treatment at E3.5/E4, the inner layer has RPE-like morphology, is pigmented, and nuclear MITF and β-Catenin protein is detected at E8 (arrowheads). Note that VSX2 protein and Collagen IX protein is not detected in this region. (L-N) Transcripts of *Pax6* and *Vsx2* are detected in the inner layer of the optic cup at E6, whereas transcripts of the RPE-specific marker *Mmp115* are restricted to the RPE and optic cup rim (arrowheads). (O-Q) Two days after BMP5 application, *Pax6* and *Vsx2* expression is downregulated in the inner layer of the peripheral optic cup, whereas *Mmp115* expression is induced in this region (arrowheads). The asterisks indicate the BMP5 bead. All expression pattern studies included *n*≥5.

Histological sections of BMP5-treated embryos revealed that high BMP5 concentrations (1-0.7 µg/µl) applied at E3.5/E4 (stage 20 to 22/23) resulted in thinning of the peripheral NR (Fig. 3H-Q; Fig. S2) and ectopic pigment granulae were observed (Fig. 3C,H). To test whether the loss of NR morphology following BMP5 exposure represented a true cell fate change, we assayed the expression of a variety of transcription, growth and differentiation factors that distinguish NR and RPE in affected embryos.

At E8 (stage 33-35), MITF protein is strongly detected in the proximal RPE (Fig. 3A′,B″) and at the optic cup rim in wild-type and PBS-treated embryos (Fig. 3F,G). No or weak MITF staining is observed in the peripheral outer optic cup, which differentiates into peripheral eye structures such as iris (Fig. 3B′,F,G). At this stage, Collagen IX and β-Catenin protein is detected in retinal tissue of the peripheral optic cup (Fig. 3B″,E-G) (Kitamoto and Hyer, 2010). In the chick, *Vsx2* and *Pax6* expression is detected in retinal progenitor cells located in the peripheral optic cup (Fig. 3L,M). Following BMP5-treatment (0.7 µg/µl), Collagen IX protein and *Vsx2*/*Pax6*-expressing retinal progenitor cells were not or sparsely detected in the peripheral margin 2-4 days after treatment (Fig. 3J,O,P; Fig. S2 and Table S1). This tissue adopted an RPE-like morphology, became pigmented, and ectopic MITF, nuclear β-Catenin and *Melanosomal matrix protein 115* (*Mmp115*) expression were detected (Fig. 3H,I,K,Q; Table S1) (Mochii et al., 1998; Iwakiri et al., 2005). Application of PBS-soaked beads at E3.5/E4 did not result in changes in gene expression (Table S1). Thus, these results indicate that BMP5 signalling can reprogram peripheral retinal cells to develop into RPE.

### BMP5 converts retinal cells into RPE in the central optic cup

**Fig. 4.**
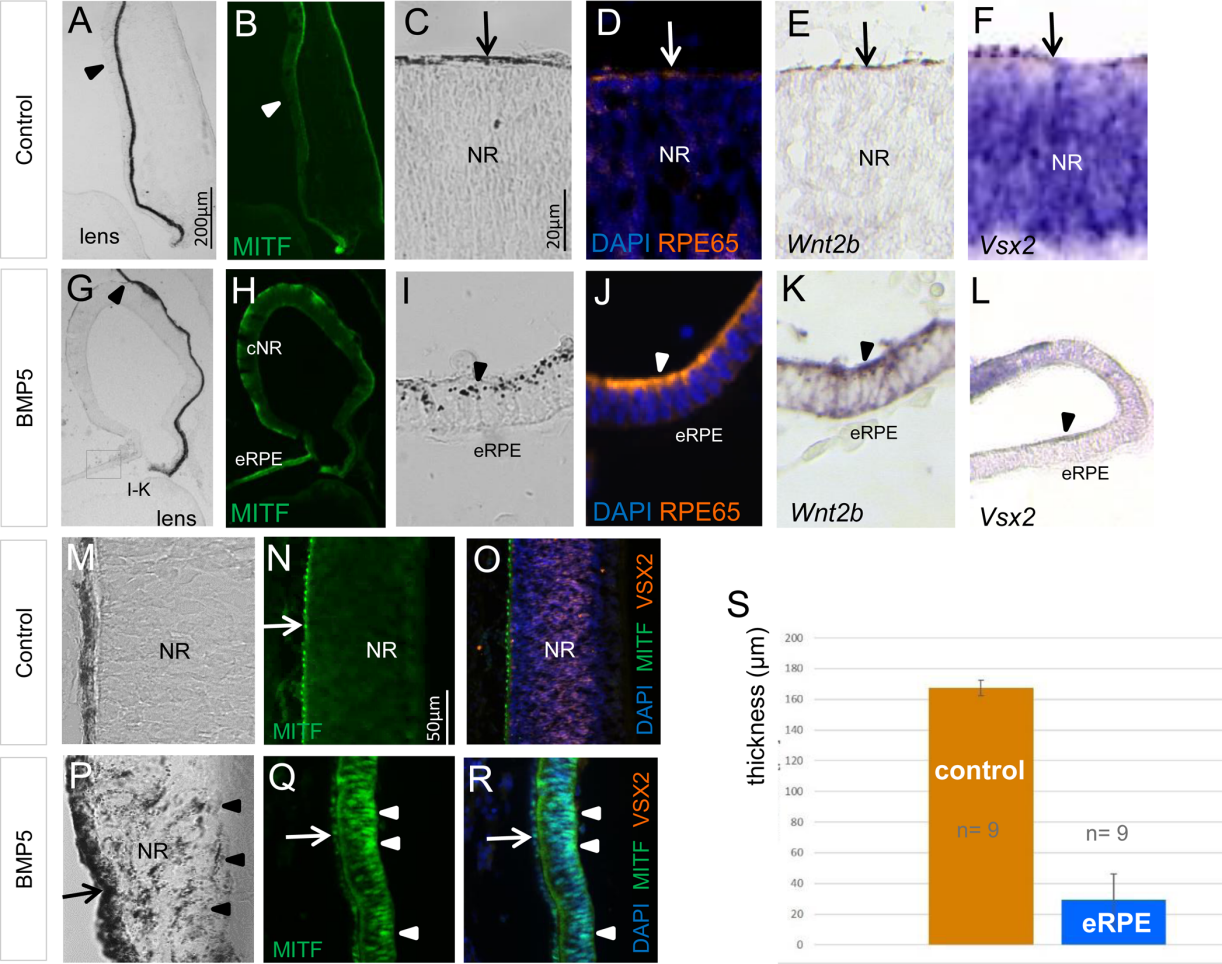
**BMP5 induces ectopic RPE development in the central NR at optic cup stages.** (A,B) Peripheral, untreated chick eye at E8 showing pigmentation and MITF expression being restricted to the outer layer of the eye. The arrowheads indicate the peripheral NR. (C-F) Higher magnification of the central chick eye at E8 showing the pigmented RPE, where RPE65 protein is detected (arrows). Faint *Wnt2b* and strong *Vsx2* expression is detected in the central NR. The arrows indicate the RPE. (G,H) Following BMP5 application at E3.5/4, the NR detached (arrowhead) and MITF protein is now detected in the central NR (cNR). Note that a part of the cNR has RPE-like morphology (eRPE). (I-L) Higher magnification images of the eRPE. Ectopic pigment granulae, RPE65 protein and strong *Wnt2b* expression are detected in this region, whereas *Vsx2* expression is downregulated (arrowheads). (M-O) Untreated chick eye at E8 showing pigmentation and MITF (arrow) and VSX2 protein distribution, restricted to the RPE and NR, respectively. (P-R) Following BMP5 application at E3.5/4, ectopic pigmentation and nuclear MITF protein are detected in the inner optic cup (arrowheads), whereas VSX2 protein is only weakly or not detected. The arrows indicate the RPE. (S) Graphical representation of the average thickness of the inner layer of the untreated (control) and BMP5-treated eye at E8. All expression pattern studies included *n*≥5.

Both BMP and Wnt signalling are required for initiating *Mitf* expression in optic vesicle cells during chick eye development (Steinfeld et al., 2013; Pandit et al., 2015). Moreover, BMP application induces ectopic *Wnt2b* expression during optic vesicle stages in the chick (Müller et al., 2007). We therefore investigated the *Wnt2b* expression pattern in treated embryos. At the time of BMP5 bead application (E4/stage 22), *Wnt2b* expression is restricted to the pigmented, peripheral optic cup and transcripts are not detected in the central and peripheral NR (Fig. 8A). Four days after BMP5 application, the central NR was considerably thinner, when compared to the contralateral or PBS-treated eye (Fig. 4I-K,S; Fig. S2 and Table S2). Pigment granulae were observed in the region, which expressed *Wnt2b* and had RPE-like morphology (Fig. 4I,K; Fig. S2). In the regions that adopted RPE-like morphology, but lacked *Wnt2b* expression, pigment granulae were absent or only sparsely observed. RPE65 is involved in the visual cycle and used as a marker for terminally differentiated RPE (Fig. 4D) (Moiseyev et al., 2005). Analysis of BMP5-treated embryos revealed that RPE65 protein was present in the central region, which had RPE-like morphology (Fig. 4J). Accordingly, *Vsx2* expression and/or VSX2 protein were not detected 3 or 4 days after manipulation (Fig. 4L,R; Fig. S2 and Table S1). Instead, we detected ectopic nuclear MITF protein in the regions that were pigmented (Fig. 4P-R; Table S1). Application of PBS-soaked beads did not result in thinning of the NR and changes in gene expression patterns were not observed (Table S1). Taken together, the results indicate that BMP5 can reprogram centrally located retinal cells to develop into RPE at optic cup stages.

### BMP5-induced cell death occurs on the basal side of the NR

**Fig. 5.**
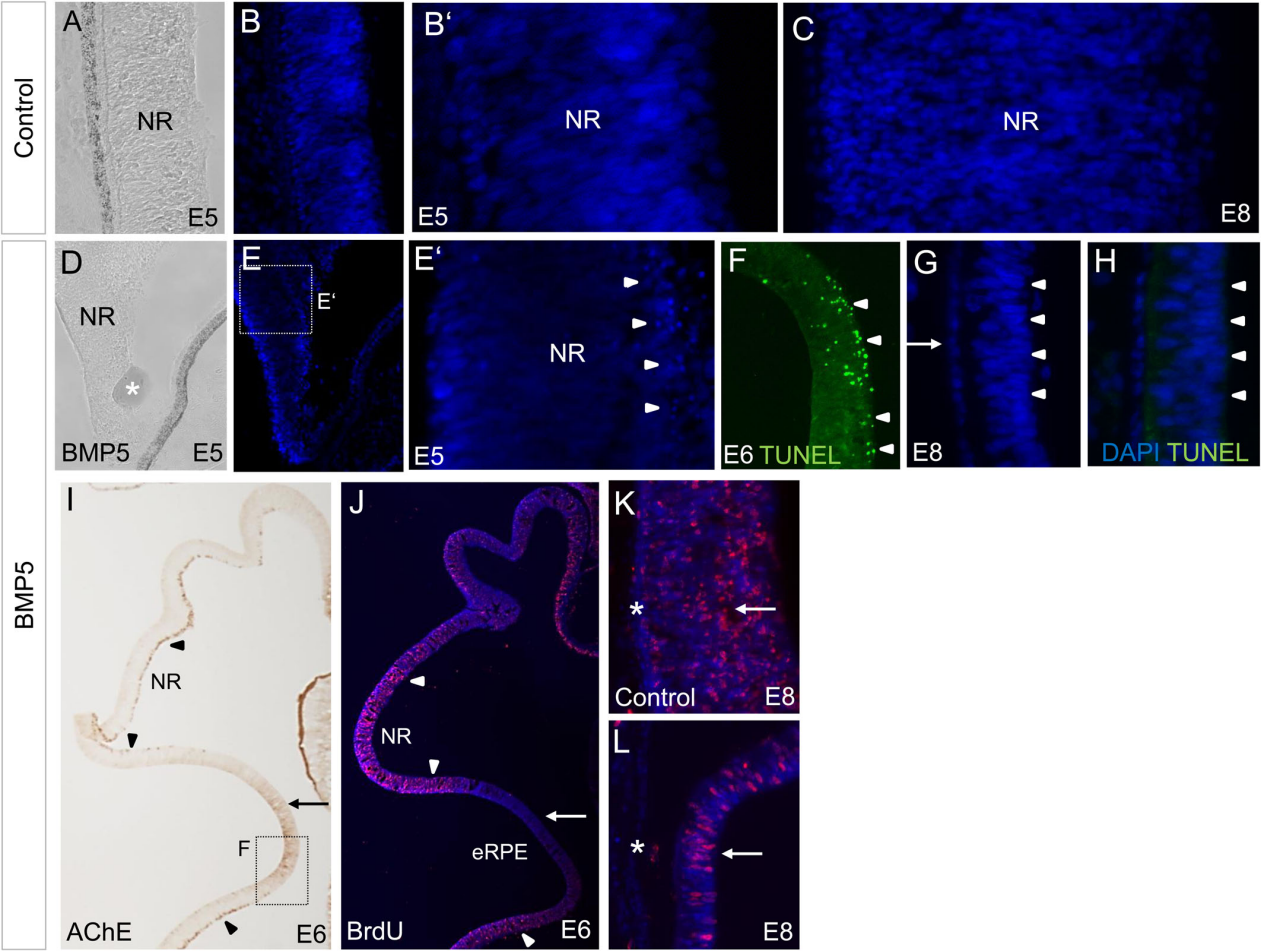
**Effects of BMP5 application at optic cup stages on proliferation and apoptosis within the inner optic cup.** (A-C) At E5 and E8, no or a single pyknotic nuclei are detected within the central NR. (D-F) Following BMP5 application, pyknotic nuclei are detected in the central NR 1-2 days after the operation. Note that pyknotic nuclei (E′) and TUNEL-positive cells (F) are dominantly found on the basal side of the NR (arrowheads) where differentiating ganglion cells are located (compare with Fig. 5A). The asterisks marks the BMP5 bead in D. (G,H) Four days after BMP-treatment, pyknotic nuclei or TUNEL-positive cells are no longer detected (arrowheads); MITF protein is still detected in this region (compare with Fig. 4Q). (I,J) In the BMP5-treated eye, the AChE-positive ganglion cell layer (arrowheads) is lost in the region which becomes re-specified (arrow). In this region, proliferation is decreased as visualised by low BrdU labelling when compared to the BrdU-positive cells of the NR (arrowheads). (K,L) BrdU labelling in the NR (arrows) and RPE (asterisks) of the contralateral and BMP5-treated eye at E8 (arrow). All cell death and BrdU studies included *n*≥3 per stage.

**Fig. 6.**
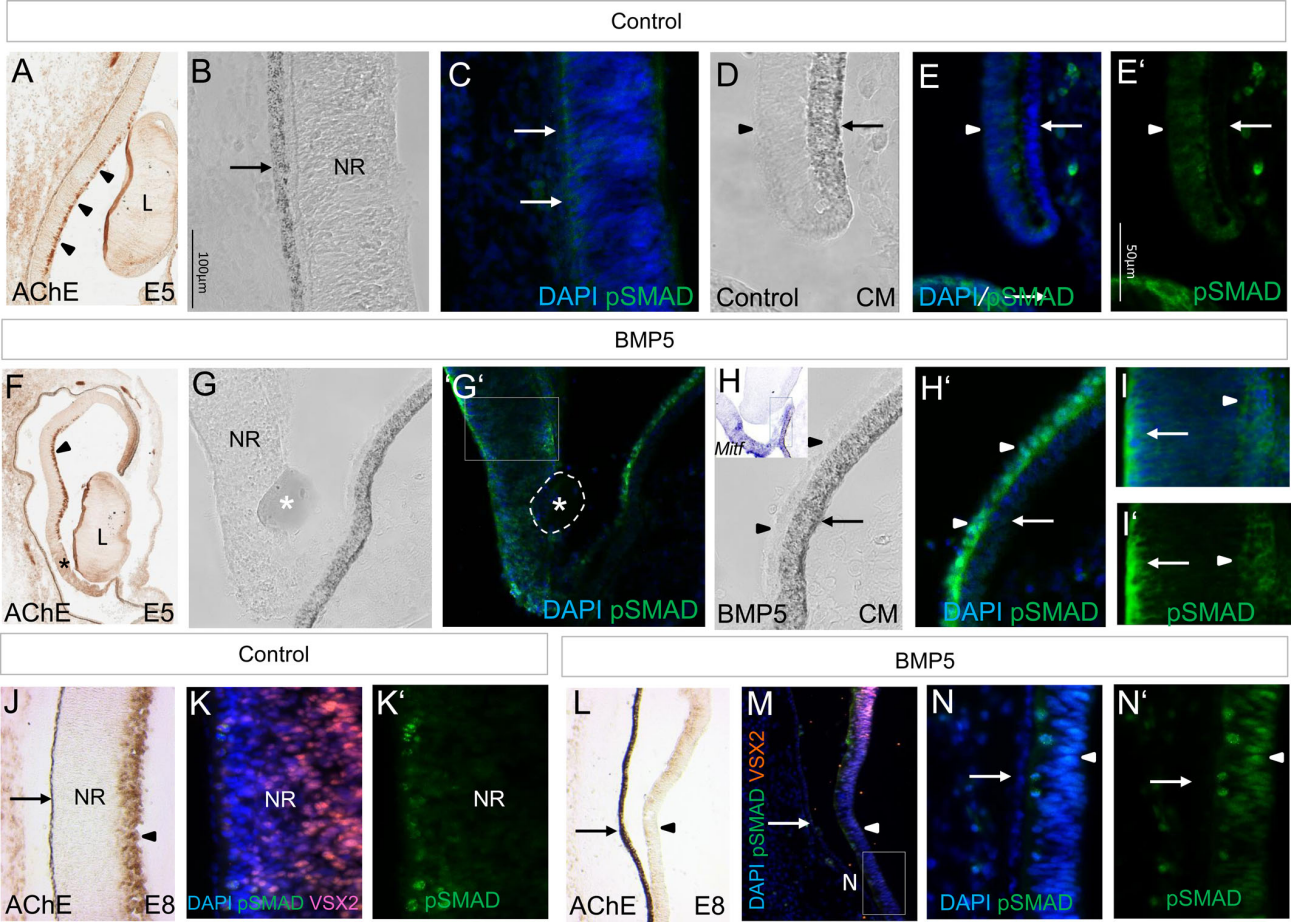
**Effects of BMP5 on pSmad1/5/8 labelling in the peripheral and central optic cup.** (A) AChE activity is detected in the ganglion cell layer of the NR at E5 (arrowheads). (B-E′) Distribution of pSmad1/5/8 in the contralateral eye 1 day after BMP5 application at E3.5/E4. Weak or no pSmad1/5/8 labelling is detected in the pigmented RPE of the central and peripheral region of the optic cup (arrows). Faint pSmad labelling is detected in the inner layer at E5 (arrowhead). (F) Following BMP5 application, AChE activity is lost in the central NR (asterisk). (G-I′) Higher magnification images in a parallel section of this region. Labelling of pSmad1/5/8 in the CM is increased (arrowheads in H′; compare to contralateral side shown in E). The inset in H shows the *Mitf* expression pattern in this BMP5-treated eye. Labelling of pSmad1/5/8 is also detected in the central NR on both the apical (arrow) and basal side. (J) AChE activity is detected in the ganglion cell layer at E8 (arrowhead). (K,K′) Higher magnification of the central optic cup showing the localisation of VSX2 and pSmad1/5/8 protein within the NR. (L-N′) Following BMP5 application at E3.5/E4, AChE activity and VSX2 protein is lost in the central NR (arrowhead in M). Labelling of pSmad1/5/8 is detected in the central NR (arrowheads; compare with Fig. 4P-R). The arrows indicate the RPE in all images. All expression pattern studies included *n*≥3 per stage.

In order to understand and characterise the process of reprogramming cells of the inner optic cup into RPE, we analysed the apoptotic state of the NR following BMP5 application. Previous studies showed that high BMP4 concentrations can induce apoptosis in the chick and mouse optic cup (Behesti et al., 2006; Trousse et al., 2001). The first postmitotic cells are observed in the basal region of the NR at the time of BMP5 application (E3.5/E4) (Willbold and Layer, 1992). To determine the level of cell death in the NR, we analysed the appearance of pyknotic nuclei and performed Terminal deoxynucleotidyl transferase dUTP nick end labeling (TUNEL) analysis. One to two days after BMP5-treatment, pyknotic nuclei and TUNEL-positive cells were detected on the basal side of the NR, whereas no cell death was detected in the NR of the contralateral eye (Fig. 5A-F; Table S1), and this corresponded with the loss of acetylcholinesterase (AChE)-positive ganglion cells (Thangaraj et al., 2012) in this region (Fig. 5I, Fig. 6F,L; Table S1). Pyknotic nuclei or TUNEL-positive cells were no longer detected 4 days after BMP5 application (Fig. 5G,H; Table S1).

To analyse the proliferative status of the NR following BMP5 application at E3.5/E4, we treated the embryos with 5-bromo-2′-deoxyuridine (BrdU) 3-5 h before fixation to identify the number of S-phases during this time interval. Two days after BMP5-treatment, AChE-positive regions of the NR were still strongly proliferative, whereas in the region that lost AChE-activity, no or few BrdU-labelled cells were detected on the basal side of the NR (Fig. 5J; Fig. S3 and Table S1). Four days after BMP5 application BrdU-positive cells were detected in both the NR of the contralateral and BMP5-treated side (Fig. 5K,L).

An indicator for active BMP signalling is pSmad1/5/8 (pSmad). In order to analyse the BMP5 effects at a cellular level, we examined the distribution of pSmad in the NR 1 day after BMP5-treatment. We detected an increase in pSmad labelling in both the peripheral and central region of the *Mitf*-positive region (compare Fig. 6B-E′ with Fig. 6G-I′). In the central region of the NR, strong pSmad labelling was observed on both the apical and basal side, where pyknotic nuclei are observed (compare Fig. 5E with Fig. 6I). Four days after the operation, we still detected pSmad labelling within the reprogrammed, MITF-positive NR (Fig. 6N,N′ and Fig. 4P-R). Application of PBS-beads at E3.5/E4 did not induce cell death, or an increase in pSmad labelling, within the NR 1 to 4 days after manipulation (Table S1). Taken together, these results indicate that BMP5 signalling induces restricted cell death in differentiating cells of the basally located central NR.

### Wnt signalling is required to convert NR into RPE

**Fig. 7.**
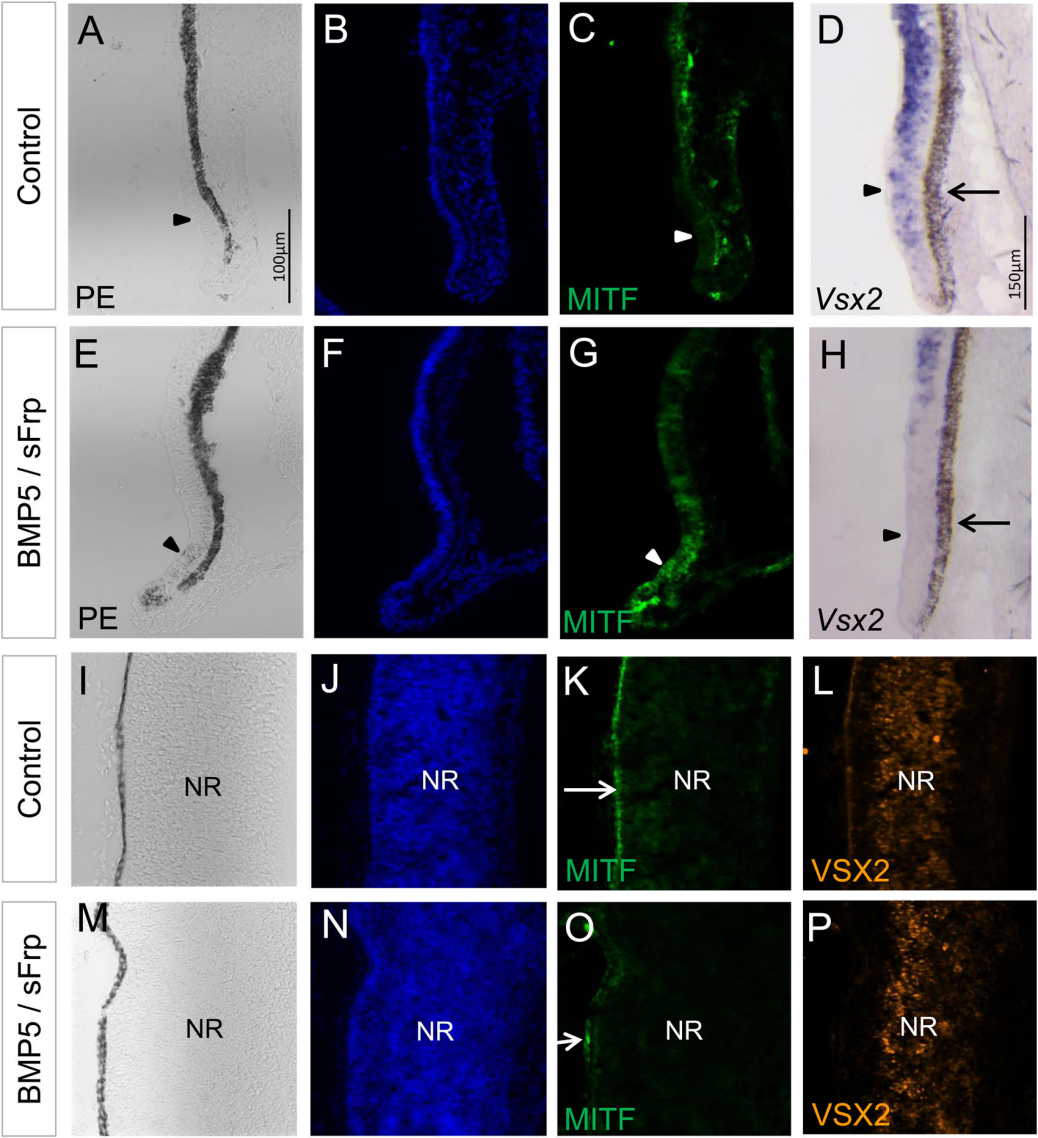
**Wnt signalling is required for BMP5-induced RPE development.** (A-C) Contralateral, untreated eye showing the absence of pigmentation and MITF protein in the inner layer of the peripheral optic cup (arrowheads). (D) *Vsx2* expression is detected in the peripheral eye cup at E8. (E-H) Following simultaneous application of a BMP5- and sFRP1-soaked bead at E3.5/E4, ectopic pigmentation and MITF expression are observed in the peripheral eye, whereas *Vsx2* expression is decreased in this region (arrowhead in H). Note that in the BMP5-treated eye, no ectopic pigmentation is observed. (I-L) Central region of the contralateral, untreated eye showing pigmentation and distribution of MITF and VSX2 protein within the RPE and NR. (M-P) In the presence of sFrp1, distribution of MITF and VSX2 protein appears to be unchanged within the NR following BMP5 application (compare with Fig. 4L,R). The arrows indicate the RPE in all images. All expression pattern studies included *n*≥5.

*In vivo* and *in vitro* studies suggest that RPE cell fate specification and hence initiation of *Mitf* expression is regulated by both BMP and Wnt signalling (Steinfeld et al., 2013; Pandit et al., 2015). Next, we tested if an active Wnt signalling pathway is required for BMP-mediated conversion of the NR into RPE. For this purpose, we implanted two beads, one soaked in BMP5 and one soaked in the Wnt inhibitor, secreted frizzled-related protein-1 (sFRP1), at optic cup stages. In the absence of Wnt signalling, BMP5 was still able to induce ectopic pigment granulae, MITF expression and downregulation of *Vsx2* expression in a few cases (Fig. 7E-H; Table S1). However, BMP5 no longer induced pigmented tissue with RPE-like morphology in the central NR. Ectopic MITF protein within the central NR was not observed and the distribution of VSX2 protein appeared to be unchanged (compare Fig. 7I-L with Fig. 7M-P; Table S1). These data show that in chick, conversion of the NR into RPE requires both the BMP and Wnt signalling pathways at optic cup stages.

### BMP5 signalling transdifferentiates RPE into NR at optic cup stages

**Fig. 8.**
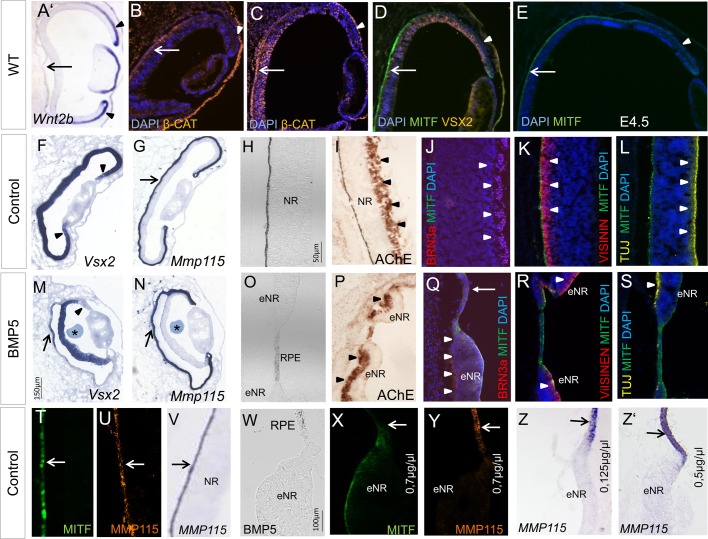
**BMP5 induces transdifferentiation of the RPE into a multi-layered NR.** (A) *Wnt2b* expression pattern at stage 22/23. Transcripts are strongly detected in the peripheral RPE (arrowheads) and are not detected in the central RPE (arrow). (B-E). Distribution of β-Catenin, VSX2 and MITF protein at E3-E4.5. Note that during these stages nuclear β-Catenin and MITF protein is strongly detected in the proximal RPE (arrows) and not or weakly in the peripheral RPE (asterisks). (F,G) Expression pattern of *Vsx2* and *Mmp115* at E4.5 in the contralateral, untreated eye. Expression is detected throughout the NR (arrowheads) and RPE (arrow) respectively. (H-J) AChE activity and Brn3α is detected in the ganglion cell layer at E8 (arrowheads). (K,L) At this stage, Visinin is detected in photoreceptor precursors apically, while TUJ1 protein is detected in the nerve fibre layer on the basal side of the NR (arrowheads). (M,N) Following BMP5 bead application (asterisks) at E3.5/E4, *Vsx2* expression is induced in the proximal region of the RPE (arrows). Note that at the same time, *Vsx2* expression is downregulated in the peripheral NR (arrowhead). One day after the operation *Mmp115* expression is still detected in the outer layer (arrows). (O-S) Loss of pigmentation in the RPE and transdifferentiation into a well-layered NR (eNR) after BMP5 application, as visualised by the pattern of AChE, Brn3α, Visinin and TUJ1 protein distribution. The newly induced NR is a mirror image of the endogenous NR shown in I-L, so that the apical to basal polarity is maintained (arrowheads). The arrow in Q marks the RPE. (T-V) Control eye showing MITF and MMP115/*Mmp115* expression within the RPE in the central optic cup (arrows). (W-Y) Following BMP5 application, the RPE has adopted NR-like morphology and MITF and MMP115 protein distribution has decreased. (Z,Z′) BMP5-induced transdifferentiation of the RPE into NR is also observed at a lower concentration (0,125–0.5 µg/µl). The arrows indicate *Mmp115* expression in the RPE, while expression is lost in the ectopic NR. All expression pattern studies included *n*≥5.

Here, we applied BMP-beads at later stages of chick eye development. At the time of BMP5 application (E3.5/E4), the peripheral and the proximal RPE can be distinguished by the distribution of signalling components of the Wnt signalling pathway and MITF protein distribution (see also Venters et al., 2015). While *Wnt2b* transcripts are detected in the peripheral RPE, nuclear β-Catenin is detected in the proximal RPE (Fig. 8A-C) (Kitamoto and Hyer, 2010). At these stages, nuclear MITF protein labelling is detected in the proximal RPE, while MITF protein is only faint or not detected in the peripheral margin at E4 to E8 (Fig. 8D,E and Fig. 3F,G). Remarkably, whereas *Vsx2* expression was downregulated in the NR 1 day after BMP5-treatment, expression was simultaneously initiated in the proximally located *Mmp115*-expressing RPE (Fig. 8M,N). Subsequently, the outer layer thickened and pigmentation, MITF and/or MMP115 protein was only faintly or no longer detected (Fig. 8O-Y). Four days after bead implantation (E8), the newly induced NR was properly layered as shown by the appearance of ganglion cell markers (AChE/BRN3α) basally and the photoreceptor precursor cell marker Visinin apically (8/8) (Fig. 8P-S). We did not observe a multi-layered NR in the peripheral *Wnt2b*-expressing RPE following BMP5-treatment (0/15) (Figs 2 and 3). Ectopic NR development in the proximal or peripheral optic cup was not observed following implantation of PBS-soaked beads at E3.5/E4 (Table S1). Thus, BMP5 application can transdifferentiate the proximal, *Wnt2b*-negative RPE to develop into a multi-layered NR.

### High BMP concentrations are required to induce ectopic RPE development

**Fig. 9.**
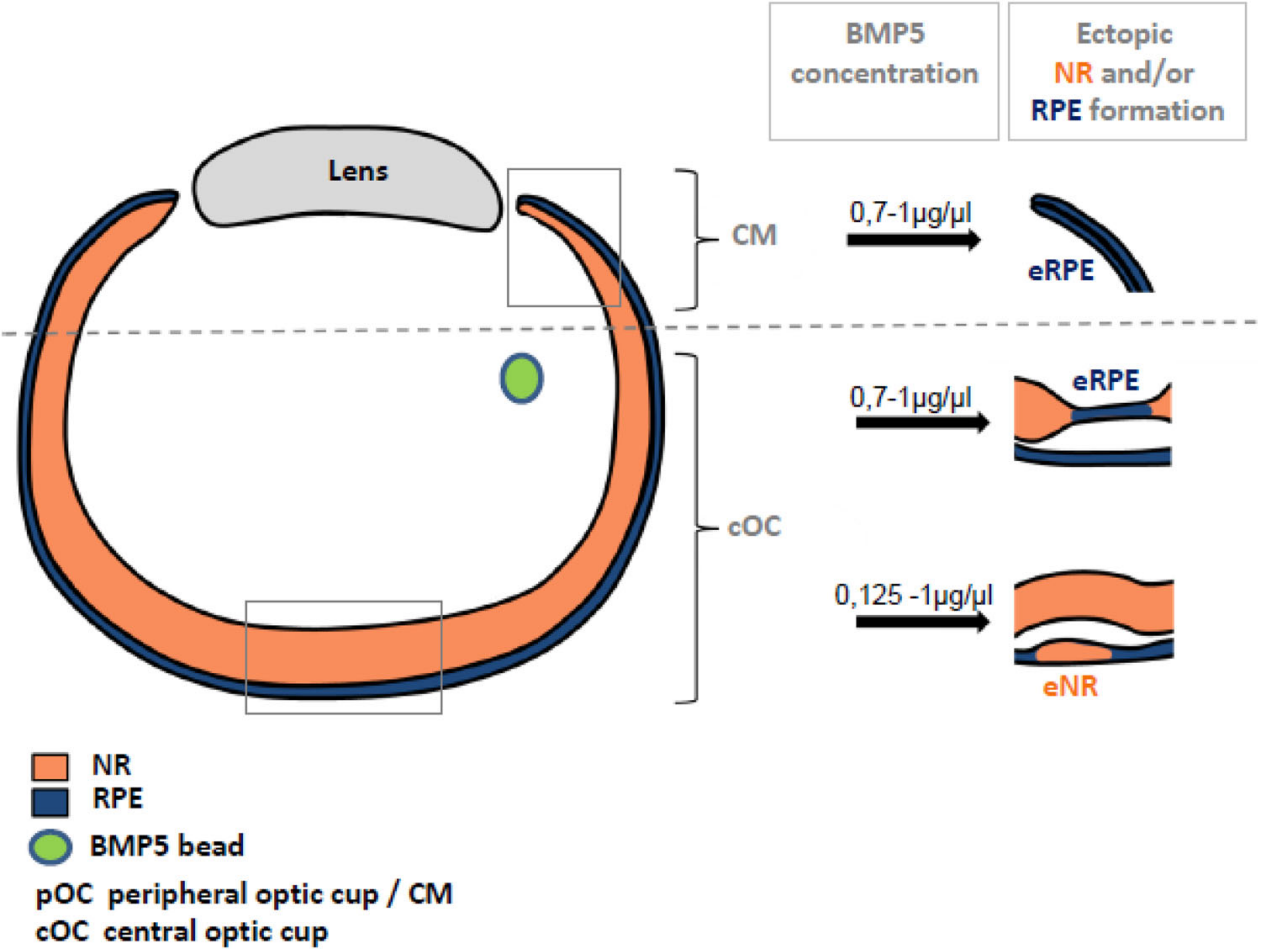
**Schematic of the dose-dependent effects of BMP-induced ectopic RPE and NR formation in the outer and inner chick optic cup.** Following application of high BMP5 concentrations (0.7–1.0 µg/µl) at E3.5/E4, thinning of the NR (orange) and ectopic *Mitf* expression in the inner optic cup is observed. At these concentrations, transdifferentiation of the proximal RPE (blue) into *Vsx2*-expressing NR is also observed. At lower concentrations (0.125–0.5 µg/µl), ectopic NR in the outer layer of the optic cup, but no ectopic *Mitf* expression in the NR, is induced. BMP5-induced *Vsx2* expression was not detected in the *Wnt2b*-expressing peripheral RPE, both at low and high BMP concentrations.

Precise levels of BMP signalling are critical for normal eye development in avian and mammalians (Behesti et al., 2006). For example, high BMP concentrations are required to induce an RPE cell fate at optic vesicle stages, while low concentrations appear to induce a retinal cell fate at these early stages (Ohkubo et al., 2002; Murali et al., 2005; Pandit et al., 2015). To test concentration-dependency at optic cup stages, we applied different concentrations of BMP5 at E3.5/E4 (1, 0.7, 0.5 and 0.125 µg/µl). While high levels of BMP5 (0.7 to 1 µg/µl) induced ectopic *Mitf* expression and pigmentation, as well as ectopic NR development (Figs 3 and 4), lowering the concentration down to 0.5 to 0.125 µg/µl no longer resulted in ectopic *Mitf* expression. However, this concentration still effectively converted RPE into NR (Fig. 8Z,Z, Fig. 9; Table S2). Thus, high BMP5 concentrations are required to convert retinal progenitor cells into RPE at optic cup stages in the developing chick embryo.

## DISCUSSION

During evolution, the eyes of different vertebrate species have adapted effectively to their environment. For example, the night-active mouse develops a rod-rich NR required for proper vision at night and therefore does not need to develop a cone-rich macula/fovea centralis. As such, during the day, visual acuity is impaired in mice when compared to birds or humans (Da Silva Souza et al., 2011; Baker, 2013). In this study, we used the developing chick embryo as an ideal model system to better understand human eye development and disease.

### High concentrations of BMP5 can convert NR into RPE in the chick optic cup

BMP and Wnt ligands and their receptors are present in the developing vertebrate eye, including the RPE (reviewed in Fuhrmann, 2008; Adler and Canto-Soler, 2007). For example, in several species, including humans, *Bmp7* transcripts are detected in the presumptive RPE at early optic vesicle and/or cup stages and in the adult RPE (Fig. S1) (Dudley et al., 1995; Dudley and Robertson, 1997; Furuta et al., 1997; Furuta and Hogan, 1998; Vogel-Höpker et al., 2000; Belecky-Adams and Adler, 2001; Trousse et al., 2001; Wistow et al., 2002; Haynes et al., 2007; Wyatt et al., 2010; Zhang et al., 2014; Huang et al., 2015). Mutations in the *Bmp7* gene can lead to anophthalmia or microphthalmia in mice and humans (Dudley and Robertson, 1997; Jena et al., 1997; Wyatt et al., 2010). In this study, we identified BMP5 as another potential signal involved in RPE development at optic cup stages during vertebrate eye development. *Bmp5* transcripts are detected in the developing RPE at optic vesicle/cup stages and we show that only high BMP5 concentrations can convert NR into RPE at later stages of chick eye development. This is the first report showing growth factor-induced formation of ectopic RPE tissue in vertebrates at optic cup stages (reviewed in Belecky-Adams et al., 2008; Spence et al., 2004, 2007b). BMPs are known to act as morphogens regulating the expression of genes in a concentration-dependent manner during vertebrate development (Murali et al., 2005; Behesti et al., 2006; Bandyopadhyay et al., 2013). For example, high BMP concentrations (>100 µg/ml) appear to induce an RPE cell fate in optic vesicle cells (Golden et al., 1999; Hyer et al., 2003; Ohkubo et al., 2002; Müller et al., 2007; Kobayashi et al., 2010; Steinfeld et al., 2013). In contrast, a low dose of BMPs is not sufficient to induce pigmentation or RPE-specific gene expression at optic vesicle stages (Fuhrmann et al., 2000; Ohkubo et al., 2002; Pandit et al., 2015), and instead an NR cell fate is induced (Haynes et al., 2007; Pandit et al., 2015; Kuwahara et al., 2015; for review see also Layer et al., 2010). That a low dose of BMPs is involved in inducing *Vsx2* expression in the mouse optic vesicle has already been suggested (Murali et al., 2005). Consistent with this, we find that low BMP5 concentrations can still transdifferentiate RPE into NR. In accordance with this, during early stages of mouse and chick eye development, *Bmp5* and *Bmp7* expression is stronger in the presumptive RPE, whereas low expression of these genes is observed in the presumptive NR (Fig. 1A) (Dudley and Robertson, 1997). In accordance with the findings that BMPs can induce both *Vsx2* and *Mitf* expression in multipotent optic vesicle cells, it has now been shown that BMP signalling can induce both *Vsx2*-expressing retinal progenitor cells (Kuwahara et al., 2015) and *Mitf*-expressing progenitor cells in pluripotent stem cells (Choudhary et al., 2017).

### Wnt signalling is required for BMP-induced RPE development at optic cup stages

Loss of function studies indicated that BMP and/or Wnt signalling is required for RPE development in chick and/or mouse (Adler and Belecky-Adams, 2002; Müller et al., 2007; Fujimura et al., 2009, 2015; Westenskow et al., 2009; Bharti et al., 2012; Steinfeld et al., 2013; Capowski et al., 2016). However, loss of BMP signalling at optic vesicle/cup stages did not appear to disturb RPE development in the mouse (Murali et al., 2005). During chick eye development, a cross-talk between the BMP and Wnt signalling pathways is required to specify the RPE in optic vesicle cells (Steinfeld et al., 2013; Pandit et al., 2015). Here, a Wnt/β-Catenin-independent signalling pathway appears to be involved in initiating *Mitf* expression (Steinfeld et al., 2013; Hägglund et al., 2013). At later stages, when the RPE differentiates, the canonical Wnt/β-Catenin pathway is involved in regulating the expression of genes required for pigment synthesis (Fujimura et al., 2009, 2015; Westenskow et al., 2009; Bharti et al., 2012; Steinfeld et al., 2013; Hägglund et al., 2013; Leach et al., 2015; Fujimura, 2016). Here, we show that at optic cup stages both the BMP and Wnt pathways are required to reprogram the NR into RPE in the chick. In the absence of Wnt-signalling, BMP signalling was not sufficient to induce hyperpigmentation and the conversion of the NR into RPE (Fig. 7). Moreover, the appearance of pigment granulae within the BMP5-induced ectopic RPE was restricted to the area in which *Mitf* and *Wnt2b* were co-expressed (Fig. 4). Indeed, a recent study showed that during the process of RPE regeneration in the mouse eye, *Wnt2b* expression was several folds increased when compared to other Wnt family members. These authors suggested that in the regenerating mouse eye, this Wnt family member might be initially involved in the process of RPE specification (Han et al., 2015).

### BMP5 induces apoptosis in differentiating NR cells and reprograms proliferating NR cells to develop into RPE

In this study, we show that BMP5 regulates proliferation and cell death, thereby inducing an RPE-like morphology within the optic cup. Following BMP5 application, cells of the central NR excited the cell cycle in regions which were reprogrammed to develop into RPE (Fig. 5J). MITF is known to regulate proliferation and differentiation of the avian and mammalian RPE (Tsukiji et al., 2009; Capowski et al., 2014). For example, following transfection with *Mitf*, labelling of BrdU-positive cells within the NR decreases, whereas dominant-negative *Mitf*-transfected cells of the RPE re-entered the cell cycle (Tsukiji et al., 2009).

At the time of BMP5 application (E3.5/E4), the first postmitotic, differentiating cells are ganglion cells detected on the basal side of the NR (Willbold and Layer, 1992). BMP5 leads to restricted apoptosis within the basal region of the NR at optic cup stages (Fig. 5D-F) (Behesti et. al., 2006; Trousse et al., 2001), as shown by the temporal presence of pyknotic nuclei and TUNEL-positive cells. Accordingly, AChE-positive ganglion cells are no longer detected in the central NR, which becomes reprogrammed into RPE (Fig. 6I). The observation that an increase in pSmad labelling is detected on both the basal and apical side of the NR suggests that BMP5 signalling affects cells of the central NR differently. BMP5-induced *Mitf* expression in proliferating, undifferentiated cells (see above), which are mainly located on the apical side of the NR, induces these cells to exit the cell cycle. This, together with the BMP5-induced cell death of differentiating NR cells, results in thinning of the NR (Fig. 5). On the other hand, MITF-positive, undifferentiated NR cells become re-specified and subsequently start to differentiate. These cells become pigmented, express a variety of differentiation markers, such as MMP115 and RPE65, and the region adopts an RPE-like morphology (Fig. 4). These results suggest that BMP-induced RPE development in the chick embryo does not result from transdifferentiating NR cells, but rather from a re-specification of undifferentiating retinal cells.

### BMPs can induce transdifferentiation of the RPE into NR

In this study, we show for the first time that BMP signalling can transdifferentiate proximal RPE to develop into a multi-layered NR (Fig. 8). This is not surprising, as BMP signalling is involved in specifying both RPE and NR at optic vesicle stages in mouse and chick (see below). During early stages of vertebrate eye development extrinsic signals released from the surrounding tissues are involved in patterning the optic vesicle into an NR and RPE domain. FGFs released from the surface ectoderm have been the prime candidates to induce NR development during vertebrate eye development (reviewed in Fuhrmann, 2010). However, recent findings suggest that BMP family members are involved in RPE and NR cell fate specification both *in vitro* and *in vivo* (Murali et al., 2005; Huang et al., 2015; Kuwahara et al., 2015; Pandit et al., 2015; Choudhary et al., 2017; Layer et al., 2010). Indeed, in the chick and mouse, interfering with BMP signalling at optic vesicle or optic cup stages disrupts both NR and RPE development (Adler and Belecky-Adams, 2002; Murali et al., 2005; Müller et al., 2007; Steinfeld et al., 2013; Huang et al., 2015). In agreement with these observations, we now show that BMPs can induce both the conversion of the NR into RPE and transdifferentiation of the RPE into NR (Figs 4 and 8). Interestingly, while BMP5 application induced *Vsx2* expression in the proximal RPE, a downregulation of *Vsx2* expression within the NR was simultaneously observed (Fig. 3P and Fig. 8M). These different and even contrasting cellular outcomes might be best explained by the interplay of the BMP signalling pathway with other signalling pathways, whereby these interactions can be either synergistic or antagonistic (reviewed in Bandyopadhyay et al., 2013). For example, at the time of BMP application around E4, *Wnt2b* expression is restricted to the pigmented peripheral eye margin and not observed in the proximal RPE (Fig. 8A) (Jasoni et al., 1999; Kubo et al., 2003; Kitamoto and Hyer, 2010). Thus, it is possible that BMP signalling in the presence of WNT2b might induce an RPE cell fate in the peripheral eye, whereas in the WNT2b-negative proximal RPE a conversion into NR is observed (Figs 8 and 9) (Venters et al., 2015).

In summary, BMPs can induce both ectopic RPE and NR in specified or committed cells of the chick optic cup, whereby the presence of Wnt-signalling favours the development of ectopic RPE formation. Our study not only establishes an ideal system to study the molecular network regulating RPE and NR regeneration, but may also in the future contribute to the generation of sufficient, functional RPE and NR cells required for clinical applications. Indeed, recent studies have shown that BMPs can induce both *Mitf* (Choudhary et al., 2017) and *Vsx2* (Kuwahara et al., 2015) expression in human embryonic and induced pluripotent stem cells. It will now be interesting to see whether the combinations of different BMP and/or Wnt family members and the concentrations used might improve the derivation of a homogenous and functional RPE or NR population.

## MATERIALS AND METHODS

### *In vivo* manipulations of the developing chick embryo

Fertilized chicken eggs (Gallus gallus, Linnaeus; Dieburg, Germany) were incubated at 38°C until E3-5 or stages 17-27 according to Hamburger and Hamilton (1951). Embryos manipulated at E3.5-E4 are usually around stage 20-24; embryos manipulated around E4.5-E5 are around stage 25-27, while embryos at embryonic day E5.5-E6.5 are around stages 29-30. Agarose beads (AffiGel Blue Gel beads, Bio-Rad) were soaked in BMP5 protein (0.125–1 µg/µl, R&D Systems, Minneapolis, USA), human sFrp-1 protein (1–2 µg/µl, R&D Systems). A small incision was made into the ventrally located optic fissure to prevent damage of the NR and RPE. One or more beads (see below) soaked in the above mentioned solutions were transferred into the egg and inserted through the slit into the optic cup. The eggs were sealed and left to develop at 38°C until they reached the desired stage. Embryos were fixed in 4% paraformaldehyde in PBS at 4°C for 2-96 h and processed as previously described (Steinfeld et al., 2013). For proliferation and cell death studies, 50 µl BrdU (25 mM) solution was injected over the manipulated chick head 3-5 h before fixation. TUNEL was performed by using an *in situ* cell death detection kit (Promega, Madison, USA). Control experiments were carried out by implanting one or more PBS-soaked beads into the optic cup of the chick embryo according to the same protocol. The manipulation itself did not appear to affect eye development as pigmentation and RPE- or NR-specific gene expression appeared to be unchanged 1-4 days after the operation (Table S1). The contralateral, untreated eye (control) is mainly represented in the figures to circumvent differences in staging and staining procedures due to the dynamic expression patterns of genes during these early stages of embryonic development. Number of cases per total is indicated as (X/Y in Tables S4 and S5). For quantitative analysis of BMP5 induced hyperpigmentation within the RPE we used densitometrical features of ImageJ. We placed at least 3 equal squares (33,5 µm2) in each of the central and peripheral RPE and determined the pixel intensity by measuring the integrals of the correlating plot diagrams. To avoid aberrations through picture dependent backlight an additional measurement was placed in a tissue free area and the values were substracted from the measurements obtained within the RPE. The total mean of the central and peripheral measurements were taken and compared with the contralateral, untreated eye. Statistical analysis was performed using the unpaired *t*-test. Data are presented as mean±standard deviation.

### *In situ* hybridisation and immunohistochemistry

RNA *in situ* hybridisation (ISH) of cryostat sections was performed as previously described (Reissmann et al., 1996). Antisense RNA probes specific for chicken *Pax6*, *Mitf*, *MMP115*, *Vsx2*, *Wnt2b* (Müller et al., 2007) were used. For immunohistochemical studies, antibodies recognizing MITF (HPA003259, Sigma-Aldrich), pSMAD1/5/8 (9516, 9511S, Cell Signaling Technologies), PY489-β-Catenin (DSHB, Iowa, USA), MMP115 (Makato Mochii, Hyogo, Japan), Retinal pigment epithelial-specific protein 65 kDa (4018BD113D9, Novus Biologicals, Littleton, USA), VSX2 (Thermo Fisher Scientific), BRN3α (Eric Turner, University of California, San Diego, USA), Visinin, TUJ1, Collagen IX and G3G4 anti-BrdU (DSHB) were used on cryostat sections (Steinfeld et al., 2013). AChE activity was visualized according to the methods described by Karnovsky and Roots (1964). Images were taken with a Stereomicroscope Nikon H550L, Axiovert S1002 and Axiophotx10 Observer D1 (Carl Zeiss) and processed with Adobe Photoshop CS5 (Adobe Systems).
